# Genetic variations of *ND5* gene of mtDNA in populations of *Anopheles sinensis* (Diptera: Culicidae) malaria vector in China

**DOI:** 10.1186/1756-3305-6-290

**Published:** 2013-10-08

**Authors:** Abdelrafie M Makhawi, Xiao-Bo Liu, Shu-Ran Yang, Qi-Yong Liu

**Affiliations:** 1 Department of Vector Biology and Control, State Key Laboratory for Infectious Disease Prevention and Control, National Institute for Communicable Disease Control and Prevention, Chinese Center for Disease Control and Prevention, Beijing 102206, China; 2 WHO Collaborating Centre for Vector Surveillance and Management, Beijing 102206, China; 3 China CDC Key Laboratory of Surveillance and Early-Warning on Infectious Disease, Chinese Center for Disease Control and Prevention, Beijing 102206, China; 4 Department of Biotechnology, College of Applied and Industrial Sciences, University of Bahri, Khartoum, Sudan; 5 Collaborative Innovation Center for Diagnosis and Treatment of Infectious Diseases, Hangzhou, China

**Keywords:** *Anopheles sinensis*, Population genetics structure, mtDNA, *ND5* gene

## Abstract

**Background:**

*Anopheles sinensis* is a principal vector for *Plasmodium vivax* malaria in most parts of China. Understanding of genetic structure and genetic differentiation of the mosquito should contribute to the vector control and malaria elimination in China.

**Methods:**

The present study investigated the genetic structure of *An. sinensis* populations using a 729 bp fragment of mtDNA *ND5* among 10 populations collected from seven provinces in China.

**Results:**

*ND5* was polymorphic by single mutations within three groups of *An. sinensis* that were collected from 10 different geographic populations in China. Out of 140 specimens collected from 10 representative sites, 84 haplotypes and 71 variable positions were determined. The overall level of genetic differentiation of *An. sinensis* varied from low to moderate across China and with a *F*_ST_ range of 0.00065 – 0.341. Genealogy analysis clustered the populations of *An. sinensis* into three main clusters. Each cluster shared one main haplotype. Pairwise variations within populations were higher (68.68%) than among populations (31.32%) and with high fixation index (*F*_ST_ = 0.313). The results of the present study support population growth and expansion in the *An. sinensis* populations from China. Three clusters of *An. sinensis* populations were detected in this study with each displaying different proportion patterns over seven Chinese provinces. No correlation between genetic and geographic distance was detected in overall populations of *An. sinensis* (R^2^ = 0.058; *P* = 0.301).

**Conclusions:**

The results indicate that the *ND5* gene of mtDNA is highly polymorphic in *An. sinensis* and has moderate genetic variability in the populations of this mosquito in China. Demographic and spatial results support evidence of expansion in *An. sinensis* populations.

## Background

*Anopheles sinensis* is a principal vector of malaria in many regions in China along with *An. lesteri*, *An. minimus,* and *An. dirus*[1,2]. *An. sinensis* has also been identified as the major malaria vector in many parts of other Asian countries, such as in Indonesia, South Korea, Thailand and Japan [3]. In China it was reported that 0.006-2.58% of *An. sinensis* was infected with *P. vivax*. The infection rate of *An. sinensis* in Taiwan was 0.02 % during 1947-1949 [4]. However, malaria outbreaks and re-emergences in China occurred only in the areas where *An. sinensis* is the principal vector in recent years [5]. Malaria was historically epidemic in the Huang-Huai River region of central China and the total malaria cases in these areas were 21.99 million, accounting for 91.2% of the total reported cases in the country in 1970s. With active implementation of malaria control measures for more than 30 years, considerable success had been achieved and the cases decreased dramatically and many counties in Huang-Huai River region reached the goals of the malaria elimination (the incidence is below 1/10,000). However, in the past decade, malaria has re-emerged in these areas, especially the Anhui Province, where a total of 26,873 malaria cases, 108,594 suspected cases and 23 deaths were reported by the annual case reporting system in 858 counties of 22 Provinces in 2008. The annual incidence was 0.21/10,000. Although, the re-emergence of malaria in central China was controlled in 2008, the number of malaria cases and the incidence of the disease in this region still accounts for 68% of the total cases reported in the country [6].

Although great success has been achieved since the launch of National Malaria Control Programme in 1955, malaria remains a serious public health problem in China [7-9]. Falciparum malaria, the most deadly among the four main types of human malaria, accounted for 14.9% of all blood-test confirmed malaria cases in 1998 [10]. Falciparum malaria had been endemic in fifteen provinces of China in the early 1950s. The endemic area of falciparum malaria was restricted to eight provinces by 1980, and only to two provinces, Yunnan and Hainan, by 1998 [10]. Consequently, knowledge regarding the distribution of this disease vector, the genetic characteristics of its populations in relation to local environmental conditions is valuable for malarial control.

*An. sinensis* belongs to Hyrcanus group that includes approximately 30 morphologically indistinguishable species. In China, 25 taxa, including synonyms, have been recorded [11]. The study on the group in China can be traced back to the 1930s. Yao and Wu [12] and Yao and Ling [13] observed that *An. sinensis* could be separated into different forms on the basis of the egg deck width. Baisas and Hu [14] described the form with narrow decked eggs as a new species, *An. lesteri.* Ho *et al*. [15] also reported *An. lesteri* in China, but they noticed some distinct bionomic characters found in China as compared to those of the Philippine form. Particularly, the Chinese form of *An. lesteri* has a strong preference for human blood [15].

Cytogenetic studies have revealed two karyotypic forms, A (XY1) and B (XY2), in *An. sinensis*[16], which correspond to distinct sequences of the second internal transcribed spacer (*ITS2*) region [17]. Both forms were present in Thailand [16], but only form B was observed in China and Korea [17,18]. Analyses of microsatellites loci revealed high polymorphic nature of the *An*. *sinensis* populations in China, which indicates that *An*. *sinensis* could be divided at least into two populations [19]. Recent molecular studies suggested that *An. lesteri* from Korea and Japan, *An. lesteri* from China and *An. lesteri* from the Philippines were all the same species [20]. Ree *et al*. [21] reported an unknown *Anopheles* species that was morphologically identical to *An. sinensis*, and Li *et al*. [22] also observed two unknown species that were morphologically similar to *An. sinensis.* Rueda [23] designated these two species as new species, *An. belenrae* sp. Nov. and *An. kleini* sp. Nov, based on their same morphologically identity with *An. pullus* and *An. sinensis*, respectively. Morphological identification of *sinensis* complex is extremely difficult, so that some members of *An. lesteri*, *An. pullus* (including the form *yatsushiroensis*) and two species (at least) are mixed in the population of *An. sinensis* in Korea.

Various levels of population subdivision of the anopheline mosquito species have been observed from nearly panmictic across a wide geographic range to highly divergent within a short distance. A high rate of recurrent gene flow and/or recency of population expansion has made the populations of some anopheline species hardly differentiated [24-26]. However, other species are composed of highly structured populations, due primarily to geographic barriers, such as mountain chains or arid valleys [27-29]. These genetic data are of special value since it may enable planning of effective strategies for malaria control [30]. For instance, the degree of dispersal of insecticide resistant individuals or genetically modified mosquitoes may provide insight for the development of novel mosquito control strategies. Therefore, understanding the genetic structures of vector species may contribute not only to predict the spread of genes of interest (such as insecticide resistant or refractory genes), but also to identify heterogeneities in disease transmission due to distinct vector populations.

A recent microsatellite study revealed two genetic pools indicating the coexistence of two genetic units in the sampled sites [31]. In this study moderate genetic differentiation was identified in the *An. sinensis* populations in China and the population divergence was not correlated with geographic distance or barrier in the range [19]. A better knowledge of the status of genetic structure in the local *An. sinensis* populations should benefit malaria control programs in China and enable more appropriate control strategies to be developed. Unfortunately, there is very limited information about *An. sinensis* population genetics.

Mitochondrial DNA remains one of the most powerful and reliable tools for detecting population structure and inferring population differences due to its high and rapid mutational rate compared with nuclear DNA [32]. Interestingly, it has been found that within mtDNA, there are regions that diverge rapidly, while other regions that are highly conserved, making the different regions suitable for analysis of different taxonomic levels [33,34]. Mitochondrial *ND5* gene was previously used as a powerful tool in elucidating the level of genetic and phylogenetic divergence between closely related species [35]. Herein, we provide data that describe/discuss the population structure, genetic variability and gene flow among *An. sinensis* populations from China based on NADH of dehydrogenase subunit 5 (*ND5*) gene of mitochondrial DNA (mtDNA).

## Methods

### Mosquito collection and identification

**Figure 1:**
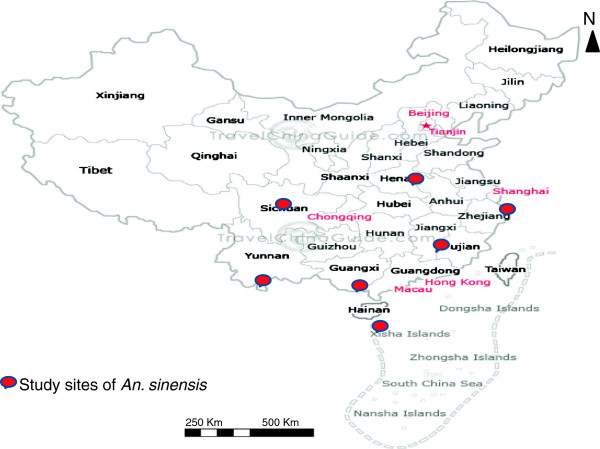
China map shows study sites.

*An. sinensis* mosquitoes used in this study were collected during the period of July 2010 to September 2012 by light traps from ten sites located in seven provinces in China (Table 1 and Figure 1). These sites are highly diverse in environmental conditions, malaria incidence, and mosquito composition and density. Central and northern Chinese provinces (e.g. Henan) are characterized by a short summer period (June - August). The southern and south western provinces (e.g. Yunnan and Hainan) are identical to subtropical areas, experiencing relatively high temperature. There is a significant threat of malaria mortality and morbidity in some Chinese provinces such as Yunnan, Hainan and Henan.

**Table 1:** **Collection data of *An. sinensis *** **populations in China**

**Population (code)**	**Collection site**	**Collection date**	**n**	**Coordinats**
Hainan (HA)	Qiongzhong	July-August, 2011	09	19°05N, 109°83E
Henan (HE-N)	Yongcheng North	Nov., 2010	09	33°42N, 115°58E
(HE-S)	Yongcheng South	Oct., 2010	19	34°18N, 116°39E
Jiangxi (JX-N)	Nanchang	July 2011	15	28°68N, 115°89E
(JX -J)	Jingdezhen	August 2011	12	29°3N, 117°22E
Guangxi (GX)	Guilin	July, 2011	10	25°29N, 110°28E
Shanghai (SH)	Baoshan	August 2011	15	31°41N, 121°48E
Sichuan (SC-C)	Chengdu	August 2011	07	30°67N, 104°06E
(SC-N)	Nanchong	August 2011	17	33°94N, 106°02E
Yunnan (YN)	Gaoligong Mountain	September 2012	27	25°11N, 98°40E

n = sample size.

*An. sinensis*, obtained from different locations, were morphologically identified using keys described by Lu *et al.*[36]. The identification was confirmed by specific-species PCR based on rDNA *ITS2*, according to the Ma *et al.*[37] protocol. Total genomic DNA for individuals used in the study were isolated using extraction kit (QIAGEN, QIAamp DNA Mini Kit) following the manufacturer’s instructions.

### Primer design, genomic DNA isolation, amplification and sequencing of rDNA *ITS2* and *ND5* of mtDNA fragments

A forward and reverse primer pair (5’-TGTGAACTGCAGGACACAT-3’ and 5’-GTTCTA CGGGCCTATCACC-3’), corresponding to *ITS2* that was invariably conserved in *An. sinensis* regardless of the region of collection, was synthesized. This primer pair was used to amplify *ITS2* fragment from genomic DNA samples followed by sequencing the amplified DNA fragment to verify the collected individual mosquitoes as true *An. sinensis*. Another forward and reverse primer pair (5’-TTGCGCCTAATCCTGCTAT-3’ and 5’-TGATTTGTGGTGTCAATGT-3’) were designed and synthesized for subsequent amplification of *ND5* DNA fragments from *An. sinensis* genomic DNA samples. The *ND5* primers were designed based on highly conserved regions of all available mosquito *ND5* sequences from GenBank databases. *Primer 3* software program was used to adjust the annealing temperature of two primer pairs. The primer pairs were used to amplify *ND5* DNA fragments from genomic DNA samples extracted from *An. sinensis* collected from different regions to assess the polymorphism of the *ND5* gene.

The PCR was conducted in a 50 μL reaction mixture containing 1 μL of a 1:200 diluted genomic DNA, 25 pmol primers, 5 μL 10X reaction buffer, 2.5 mM MgCl2, 200 μM of each dNTP, and 1 U Taq polymerase. The cycling condition was 5 minutes denaturation at 94°C, followed by 35 cycles of 15 seconds denaturation at 94°C, 15 seconds annealing at 50°C, and 1 minute extension at 72°C, ending with a final extension for 5 min at 72°C. Amplified PCR products were purified and then unidirectionally sequenced for both strands for each one of the two regions by Sangon Biotech (Shanghai Co., Ltd., Beijing, China).

### Sequences alignment and analysis

The *ND5* gene sequences from 140 *An. sinensis* individuals were aligned by using BioEdit program 5.0.9 [38]. The Haplotype diversity, its variance and nucleotide diversity [39] among populations of *An. sinensis* from different provinces and/or sites, were estimated using the program DnaSP 4.9 [40]. The Tajima [41] and the Fu and Li [42] tests were used to examine the hypothesis of selective neutrality of nucleotide substitutions. Tajima’s D test was utilized to examine whether the average number of pair-wise nucleotide differences (K) between sequences was larger or smaller than expected from the observed number of polymorphic sites (S).

Population pairwise net genetic distances based on Slatkin’s linearised *F*_*ST*_[43], and hierarchical analysis of molecular variance (AMOVA) [44,45] were estimated Using Arlequin 3.5. 1.3 [46]. The significance of F_*ST*_ evaluated was based on 1023 random permutations. Demographic analysis and spatial parameters were assessed using the distribution of pair wise sequence differences (mismatch distribution) of Rogers and Harpending [47]. We used Tajima’s D [41] and Fu’s Fs statistics [48]. The significance of the *D* and *F*s values of Tajima and Fu, respectively, was evaluated by comparison with randomly generated values based on the observed (S) with 10,000 repeats. The Mantel test [49] was performed to test significant correlation between population genetic distance and linear straight geographical distances.

A TCS software program 2.1 [50,51] was used to construct a haplotypes network and estimated genealogy relationship between haplotypes of *An. sinensis* populations collected from different sites in China.

### Ethical approval

We obtained ethical approval from the Ethical Review Committee of Chinese Center for Disease Control and Prevention (No.201214). Permission was also obtained from the directors and related departments of provincial CDC in seven provinces in China.

## Results

### *An. sinensis* identification

All specimens used in this study were confirmed as *An. sinensis* either by PCR based on species-specific *ITS2* sequences. Alignment of 30 representative *ITS2* sequences of rDNA (accession number KC769646), in conjunction with different sites of collection, confirmed that only sequences of *An. sinensis* were used.

### Characterization and Haplotypes estimation of *ND5*

A 836-bp segment of mitochondrial DNA (mtDNA), corresponding to coding region of NADH dehydrogenase gene subunit 5 (*ND5*), was successfully amplified. Analyses were carried out on 729 bp of mtDNA dehydrogenase gene subunit 5 (*ND5*) from 140 *An. sinensis* individuals collected from ten sites in seven Chinese Provinces. A total of 84 haplotypes of mtND5 were determined in *An. sinensis* individuals collected from China (GenBank accession numbers: KC565754 – KC565837), in which 71 polymorphic sites were detected within these populations. The partial nucleotide sequences of *ND5* gene showed variable polymorphic sites within and among *An. sinensis* populations. The frequencies of haplotypes clearly divided these populations into three clusters. Among them, one cluster (n = 51) had low variable sites including ancestral populations, another cluster (n = 32) displayed moderate polymorphism (at least two main constant variable sites) with nucleotide substitutions at positions (55 and 325) respectively, and the third population (n = 57) showed many variable sites with one main constant site at position 529. No characteristics of heteroplasmy and insertion or deletion events were shown within the analysed mtDNA sequence.

The other nucleotide substitutions in *ND5* gene of *An. sinensis* populations were synonymous (Additional file 1: Table S1). There were seven informative non-synonymous substituted sites with amino acids replacement. The first substitution site that corresponding to position 258 (N258S) only found in Yunnan population. Replacement corresponding to position 283 (K283N) occurred in seven populations (GX, HA, HE-N, HE-S, JX-J, JX-N and SH). Substitution at position 309 (N309S) was found in populations (JX-J, HE-S and SH). Substitutions at positions 379 and 385 (R379S; K385N), respectively, were detected in seven populations (HA, HE-S, JX-J, JX-N, SC-N, SH and YN). Substitution at position 529 (K529N) occurred in all populations except GX. Also substitution at position 687 (I687T) was detected in all sites except YN (Additional file 1: Table S1).

### Genealogical relationships among populations groups of *An. sinensis*

**Figure 2:**
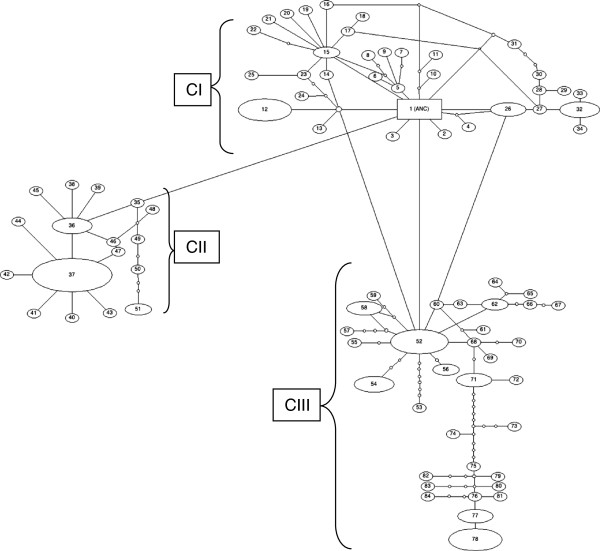
**Haplotypes network of *An. sinensis *populations collected from China based on 729bp of mtDNA *ND5 *gene.** The clusters from each other are differentiated by a single mutation step. The size of each observed ellipse indicates the proportion of the haplotype in populations, the ancestral (ANC) haplotype is rectangular. Small circle is an expected mutation. C = cluster.

The haplotypes (Additional file 1: Table S1) and genealogy network (Figure 2) revealed a network with three clusters separated by a single nucleotide differences. Each one of these networks represents group of haplotypes clustered together with common haplotype and all connected together with ancestral group. In addition to identifying unique observable haplotype frequency, the network analysis also detected the expected mutations and mutational steps that separated between haplotypes. These networks clearly illustrated the genealogical relationship between haplotypes and therefore, the illustration proposed subdivision of *An. sinensis* populations into three clusters.

### Genetic differentiation of *An. sinensis* populations

Table 2 shows the analyzed results of *An. sinensis* populations. Thus, haplotype diversities were ranged from 0.7 to 1.00; average number of nucleotide differences was 2.0 – 9.19, nucleotide diversities; and average number of mutations was 0.0027 – 0.013 and 0.002 – 0.02, respectively. The pairwise differences within the populations of *An. sinensis* were found in the range of *F*_*ST*_ = 0.0007 – 0.341 and gene flow of Nm = 0.97 – 769.5 with obviously significant difference in Yunnan province as compared to other sites (Table 3).

**Table 2:** **Haplotypes and nucleotide diversity of *An. sinensis *** **populations collected from China**

**Population**	**S**	**H**	**Hd ± SD**	**K**	**π ± SD**	**θ**
HA	28	9	1.00 ± 0.05	6.19	0.0128 ± 0.003	0.015
HE-N	13	9	1.00 ± 0.05	3.83	0.0053 ± 0.0007	0.007
HE-S	35	17	0.988 ± 0.02	6.56	0.009 ± 0.002	0.014
JX-J	27	12	1.00 ± 0.03	6.53	0.009 ± 0.002	0.014
JX-N	25	15	1.00 ± 0.02	6.19	0.0086 ± 002	0.011
GX	13	8	0.93 ± 0.08	3.64	0.005 ± 0.001	0.006
SC-C	4	3	0.714 ± 0.13	2.00	0.0027 ± 0.0005	0.002
SC-N	26	11	0.882 ± 0.07	5.32	0.0073 ± 0.002	0.011
SH	29	14	0.991 ± 0.03	7.44	0.010 ± 0.002	0.013
YN	25	8	0.849 ± 0.04	9.16	0.013 ± 0.001	0.009
Overall**	71	84	0.981 ± 0.004	7.17	0.0098 ± 0.0007	0.02

Tajima’s D = Not significant for all, Fu & Li’s D & F were not significant for all except for overall**. S = number of segregating sites, H = number of haplotypes, K = Average number of nucleotide differences, Hd = Haplotypes diversity, π = Nucleotide diversity, SD = standard deviation, θ = Average number of mutation per sequence. For population abbreviations see Table 1.

**Table 3:** **Genetic differentiation and gene flow between populations of *An. sinensis *** **collected from China**

**Population**	**HA**	**HE-N**	**HE-S**	**JX-J**	**JX-N**	**GX**	**SC-C**	**SC-N**	**SH**	**YN**
HA		3.75	42.43	13.76	−25.35	2.22	2.04	5.34	23.1	42.33
HE-N	0.118	-	9.61	769.5	27.3	6.12	5.03	4.6	26.83	1.53
HE-S	0.012	0.05	-	−78.93	−47.46	2.05	1.75	3.25	55.64	3.93
JX-J	0.035	0.0007	0.006	-	−18.54	3.16	2.81	4.33	22.32	3.1
JX-N	−0.020	0.018	0.011	−0.028	-	3.17	2.87	6.27	−29.24	4.9
GX	0.184	0.076	0.196	0.137	0.136	-	−10.34	−49.76	4.79	1.12
SC-C	0.197	0.090	0.222	0.151	0.149	−0.051	-	−165.83	3.16	0.97
SC-N	0.085	0.098	0.133	0.104	0.074	−0.010	−0.003	-	7.91	1.95
SH	0.021	0.018	−0.009	−0.023	−0.017	0.095	0.137	0.06	-	4.74
YN	0.011*	0.246**	0.113**	0.139**	0.093**	0.309**	0.341***	0.204**	0.095**	-

Significance of *χ*^2^ : * *P* < 0.05, ** *P* < 0.01, *** *P* < 0.001. *F*_*ST*_ = genetic differentiation between populations, *Nm* = gene flow. Numbers above diagonal are *Nm*, numbers below diagonal are *F*_*ST*._ For population abbreviations see Table 1.

Tajima’s D test of departures from the neutral expectations did not show significant deviation from neutrality. However, Fu and Li’s *D* and *F* statistics indicated a significant deviation from neutrality only for overall populations (*P* < 0.01), suggesting the existence of excessive rare nucleotide polymorphisms, with possible effects of purifying, selection, or population expansion. For other populations, all statistics tests did not reveal significant differences, suggesting that the nucleotide substitutions of the *ND5* gene are consistent with neutral evolution theory.

### Population structure of clustered groups of *An. sinensis* populations

Based on the haplotype polymorphism data of the mtDNA, the results revealed the presence of three clustered populations of *An. sinensis* in China (Additional file 1: Table S1), with similar findings supported by haplotype network (Figure 2). These results indicated the pattern of population structure within *An. sinensis* in China. AMOVA results shown in Table 4 revealed that most of the genetic variance lay within the populations (63.57 – 72.51%) than among the populations (27.49 – 36.43%). Three clustered populations had similar results, with a high proportion of the overall genetic variation being attributed within population levels (Table 4). On average, a relatively high percentage of the total genetic variation was attributable within cluster comparisons for all samples (68.68%). The same finding was confirmed by a significant genetic differentiation in comparisons at the hierarchical level among populations within clusters (*F*_*ST*_ = 0.364, 0.275, 0.332 and 0.313; *P* < 0.0000) in the three clusters or overall populations respectively.

**Table 4:** **AMOVA results among three clustered *An. sinensis *** **populations collected from China**

**Cluster**	**Source of variation**	**d. f**	**Variance components**	**Variation (%)**	**Fixation Index (*F*_ST_)**	**P-value**
I & II:	Among populations	1	0.88	36.43		
	Within populations	81	1.54	63.57	**0.364**	**0.0000**
I & III:	Among populations	1	1.21	27.49		
	Within populations	106	3.19	72.51	**0.275**	**0.0000**
II & III:	Among populations	1	1.66	33.16		
	Within populations	87	3.34	66.84	**0..332**	**0.0000**
Overall:	Among populations	2	1.26	31.32		
	Within populations	137	2.76	68.68	**0.313**	**0.0000**

d.f. = degree of freedom. Significance tests (1023 permutations).

### Demographic and spatial history of *An. sinensis* populations in China

The distribution of pairwise nucleotide differences for the total *An. sinensis* samples, revealed generally patterns, characteristic of a population that has undergone a large expansion. According to the mismatch distribution analysis, the hypothesis of spatial experienced expansion could not be rejected in the three clusters and overall samples. A good fit of the spatial expansion was also observed for all clustered samples based on Sum of Squared deviation (SSD) values that were not significant in all the cases (Table 5). With these results, our data support population growth and range expansion of *An. sinensis*. We also calculated the sum of squared deviation and raggedness index under the demographic expansion model for each cluster and found that all populations in the three clusters had low and non-significant values (Table 5).

**Table 5:** **Mismatch and neutrality tests results of three clustered *An. sinensis *** **populations collected from China**

			**Mismatch**				**Neutrality Tests**	
			**Demographic**		**Spatial**		**Tajima’s**	**Fu's**
**Cluster**	**N**	**S**	**SSD**	**Rag**	**SSD**	**Rag**	***D***	***Fs***
I	51	27	0.005(0.11)	0.033(0.17)	0.005(0.16)	0.033(0.17)	−1.3884	−26.1251****
II	32	17	0.010(0.46)	0.049(0.56)	0.010(0.33)	0.049(0.56)	−1.2669	−11.0673****
III	57	50	0.016(0.29)	0.009(0.72)	0.016(0.2)	0.009(0.72)	−0.5710	−11.3590**
Overall	140	84	0.011(0.29)	0.031(0.23)	0.011(0.23)	0.031(0.48)	−1.0750	−16.1840**

N = sample size, S = Segregating sites, SSD = Sum of Squared deviation, Rag = Harpending's Raggedness index. Significance ** *P* < 0.001, **** P < 0.0000, all = all samples together (Un-clustered). No Tajima’s *D* P-values are significant. Number in parentheses is *P* value.

**Figure 3:**
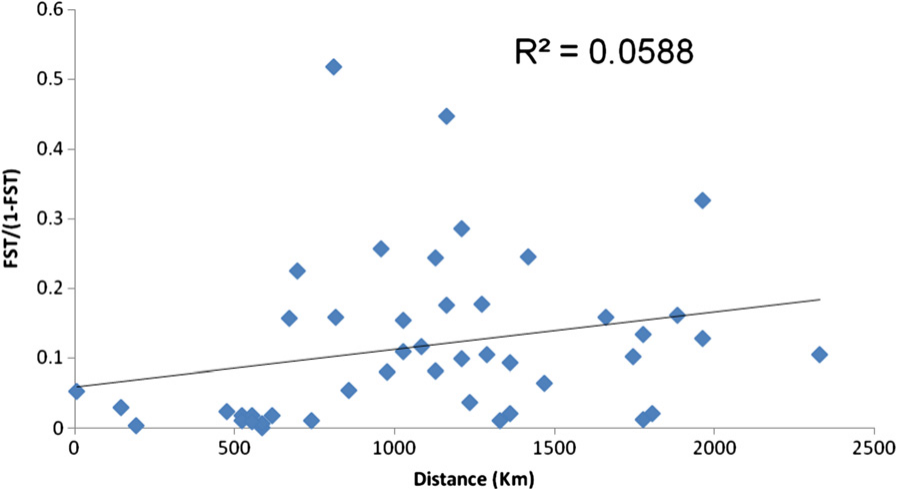
**Correlation between average *F***_***ST***_**and geographic distance between collection sites for pairwise comparisons of *An. sinensis *populations.***P* = 0.301.

Neutrality tests of Tajima's *D* revealed non-significant negative values in all individual populations of the three clusters and pooled samples (Table 5). However, negative, large and highly significant Fu's *F*s values were observed (*P* < 0.001) in the three clustered populations and pooled samples (Table 5). Fu's *F*s is considered to be more sensitive in detecting deviations from neutrality and thereby suggesting possible population expansion. The expansion may have been restricted to separate local areas that resulted in the non-significant negative Tajima's *D* values for clusters and all populations. The Mantel test results showed in Figure 3 supported the AMOVA results, that entire pooled populations lack of correlations between genetic (*F*_*ST*_) and geographic distance (R^2^ = 0.058, *P* = 0.301).

### Abundance of *An. sinensis* population groups in different Chinese provinces

**Figure 4:**
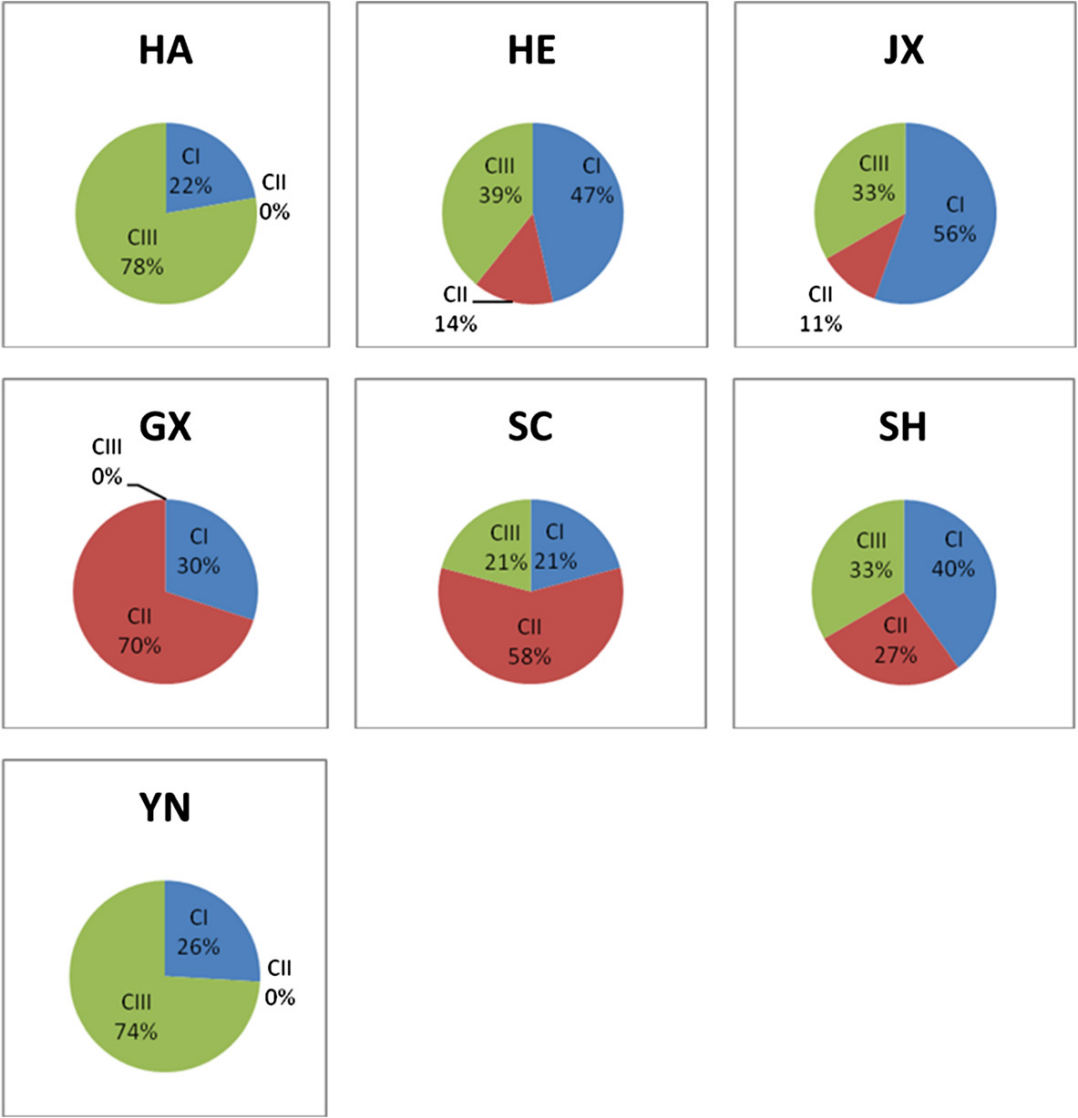
**Abundance of *An. sinensis *** **population clusters in Chinese provinces.**

Although all three clusters were observed in *An. sinensis* samples collected from different sites, there were apparent differences in relative abundance for given groups. Figure 4 illustrates the distribution of the three clustered populations of *An. sinensis* in different geographical locations in China. Hainan and Yunnan provinces showed high abundance of cluster III (78% and 74, respectively). In contrast, CII became the majority in Guangxi and Sichuan (70% and 58%, respectively). Although CI was present in all sites, it was noticeably more abundant than the other two clusters in Henan and Jiangxi (47% and 56%, respectively). Shanghai showed relatively even distribution of the three populations (Figure 4).

## Discussion

It has generally been accepted that the degree of the polymorphisms of *ND5* gene serves as a dependable representative for assessing the genetic variations in populations of a given species [35]. The present study described an analysis of 729 bp sequence of *ND5* mitochondrial DNA in *An. sinensis* populations obtained from 10 collection sites in seven Chinese provinces. The results indicate that *An. sinensis* populations in China were highly polymorphic in the ND5 gene. In most populations high level of polymorphism and moderate genetic differentiations were detected in this mosquito (*F*_*ST*_ = 0.001 – 0.341). These results are comparable to previous findings on *An. sinensis* and its member group of Hyrcanus *An. lesteri*[19,52,53]. Also these results are in agreement with *An. baimaii* in India [54], *An. darlingi* malaria vector in Central and South America [55-57], *An. albimanus*[58] in Latin America and African malaria vectors *An. gambiae*[24], *An. arabiensis* and *An. funestus*[44,59,60].

Neutrality tests of Tajima’s *D*, Fu and Li’s *D* and *F* did not detect deviation from neutrality theory in the *ND5* gene within any population of *An. sinensis*. Only Fu and Li’s *D* and *F* tests were significant when all populations from different sites pooled together, and this perhaps is due to a high level of polymorphism within *ND5* gene (71/140). These results support the neutral theory of mitochondrial gene evolution and suggested that there is a large effective population size of *An. sinensis* in these locations.

Generally, the results showed that there is low-to-moderate genetic differentiation and high gene flow within different populations except the YN population that displayed a significantly high level of genetic differentiation and restricted gene flow with all populations except Hainan population (*F*_*ST*_ = 0.011; *Nm* = 42.33). This could be due to bias of low sample size in Hainan (n = 9) but similar samples were generated from (HE-N and SC-N), that give significant results with YN. Moreover, the two sites of Hainan and Yunnan are somehow similar in their conditions for mosquito habitats. Although distantly apart, both sites belong to typical temperate tropic zones where there are great seasonal variations in abundance of mosquito populations that reach their peak during summer [61]. Yunnan is noted as centre of biodiversity [61-64]; because of it’s a highly complex region topographically due to its transitional position from tropical southern Himalayas to eastern Asia and from tropical Southeast Asia to subtropical China as well as at the junction of the India and Burmese plates, derived from Gondwanaland, and the Eurasian plate [65]. Further studies in these areas could be established by collecting and analysing more *An. sinensis* samples over several seasons. The climatic condition could impose selection on *An. sinensis* populations. In addition, *An. sinensis* breeds in a wide range of habitats including natural and artificial [4,64].

Interestingly, the results of haplotypes and genealogy revealed the presence of three clustered populations of *An. sinensis* across collection sites in China. In general cluster I approximately represented 36% (n = 51), cluster II includes relatively less samples 23% (n = 32) where 41% (n = 57) belong to cluster III. Cluster I was found in all populations collected across the study zone (ancestral), cluster two was found in 8 populations out of 10 (except HA and YN) where cluster III occured in 9 populations (except SC-C). The three clustered populations were assigned by at least one common haplotype or nucleotide substitution and/or amino acid(s) replacement. The proportion of the three clustered populations varied from site to site in China. For example, the cluster three of *An. sinensis* population is abundant in Hainan and Yunnan, but moderately abundant in Shanghai and Henan and absent in Guangxi. These results are similar to those reported in a previous study [19] that indicated that there were two gene pools found within *An. sinensis* populations collected across China from 20 collection sites (one pool included six populations and the second pool included eight populations). In that study the author reported that there was coexistence between two gene pools and specimens were assigned to one gene pool when it is remaining greater than 80%. A similar finding was reported in South Korea that *An. sinensis* includes two groups associated with the presence of a mountain functioning as a genetic barrier [52]. Normally once there are two populations possible, there is, an intermediate group. In present study cluster II could be an intermediate group. In this cluster (C II) obviously only a few individuals share polymorphic sites with each one of the other two clusters (Additional file 1: Table S1), which could be due to an ancestral background.

An AMOVA analysis using the three clusters found that 31.32% of the variance was attributed to between populations and 68.68% to within populations. A similar phenomenon of variance pattern was observed in comparing three clusters. There is substantial significant genetic differentiation among populations of the three clusters (*F*_*ST*_ = 0.313; *P* < 0.0001). The level of genetic differentiation is relatively similar to that previously detected in *An. sinensis* populations in China using microsatellites [19]. The distribution of the three clusters does not seem to be attributable to geographical range because no correlation between genetic and geographic distance was detected based on the mantel test results. Moreover, sympatric occurrence of more than one clustered populations in the same study site is further evidence that distance has no major impact on genetic differentiation. Similar results have been reported for *An. gambiae* and *An. arabiensis* malaria vectors in Africa [66]. In that report, the authors suggested that the high level of differences in these populations was likely due to some reproductive isolations rather than physical barrier or distance and also possibly due to demographic history or ecological diversification. The wide range of distributions for this mosquito population of *An. sinensis* is an importance factor for malaria control and that suggests further examining at a local level for microgeographic scales in lack of isolation by distance (IBD), for understanding population connectivity and vector dispersal across multiple spatial scales.

Mismatch distribution of demographic and spatial analyses were assessed using the distribution of pairwise sequence differences and suggested population expansion of *An. sinensis* in China. Both SSD and raggedness index supported the evidence of population expansion. Moreover, negative and significant values of Fu’s *Fs* are further indicators for population growth. These findings are also consistent with pervious reported results of *An. sinensis* in South Korea where two groups of *An. sinensis* have recently experienced expansion in population size based on mitochondrial control region [52].

Genetic diversity is a key factor that enables adaptation and persistence of a natural population towards changing or adverse environmental conditions. Its remains true that our analyses are based on a single marker, mtDNA, which may not be representative of the genome as a whole. Although most of the polymorphism in mtDNA sequences is likely to be neutral, positive or negative selection on any site within the mtDNA genome will influence the pattern of variation across the whole molecule [67]. Our results emphasize the need for further investigation with deeper sampling (especially in the areas where there are more than one cluster) using more polymorphic nuclear markers to elucidate the forces that shape and maintain the population structure. Further studies are required to investigate the three clusters of *An. sinensis* regarding ecology and susceptibility to malaria transmission.

## Conclusion

These results have implication for the interpretation of genetic population structure in *An. sinensis*. Our results revealed that the populations of *An. sinensis* in China are highly polymorphic within the *ND5* gene of mitochondrial DNA with moderate genetic variability. There are at least three clustered populations with different abundance within Chinese provinces. The occurrence of more than one sympatric population could be due to adaption of the local environmental factors such as type of breeding sites and its availability in different seasons and/or as well as for control measures. Geographic range does not seem to have a major effect on the genetic variability of *An. sinensis*. Further exploring the mechanism for the absence of a given population may provide insight towards development of new vector control strategies.

## Competing interests

The authors declare that they have no competing interests.

## Authors’ contributions

AMM performed lab experiments, data interpreting and wrote the manuscript. XBL and SRY conducted the field work and samples collection. QYL conceived and designed the research, supervised the field, lab work and wrote the manuscript. All authors read and approved the final version of the manuscript.

## Supplementary Material

Additional file 1: Table S1 Polymorphic positions of *ND5* (mtDNA) of *An. sinensis* collected from China. Click here for file
