# Supplementary material for: Genetic variations of ND5 gene of mtDNA in populations of Anopheles sinensis (Diptera: Culicidae) malaria vector in China
Source: Parasit Vectors. 2013 Oct 8;6:290. doi: 10.1186/1756-3305-6-290 (PMC4228240; doi:10.1186/1756-3305-6-290)
Supplement: Additional file 1: Table S1 — Polymorphic positions of ND5 (mtDNA) of An. sinensis collected from China. [file 1756-3305-6-290-S1.doc]

Additional file 1: Table S1. Polymorphic positions of *ND5* (mtDNA) of *An. sinensis* collected from China

14123445791111111111112222**2**222**2**22**3**3333333**33**33334444444444**5**55566666666**6**6

687685940002445677890345**5**667**8**89**0**1223346**78**89991123346899**2**38900222445**8**9

0694281627423876**8**566**3**92**9**0254794**95**81471812320706**9**85547258120**7**4

**H** **n** **Population(s)**

1 TTGTTTACAAAATAAAATTCTTGATTAAGAAACATCTTCAAAAATCTTTTACTAAATGTAATTCTCACGTT 6 3SC-C,SC-N,JX-N,GX

2 ................................................................C...... 1 HE-S

3 .....................................................T................. 1 HE-N

4 ..T......................................G............................. 1 JX-J

5 ......................................T................................ 1 JX-N

6 ......................................T.............C.................. 1 JX-J

7 ......................................T..................A..G.......... 1 JX-J

8 ....................C.........T.......T........................T....... 1 HE-S

9 ......................................T...........................G.... 1 JX-J

10 ...............................G..............................C........ 1 JX-N

11 ...............................G...................................T... 1 SC-N

12 ..........................G..............................A............. 7 7YN

13 ........................C................................A............. 1 HA

14 ..............................T..........................A............. 1 JX-N

15 ..............................T........................................ 2 HE-N, HA

16 .C............................T........................................ 1 SH

17 ..........G...................T........................................ 1 HE-S **CI**

18 ..........G...................T.............................G.......... 1 HE-S

19 ...........................T..T........................................ 1 JX-N

20 ..............................T...........G............................ 1 JX-J

21 ..............................T.............C.......................... 1 HE-S

22 ..............................TG.....A................................. 1 GX

23 ..............................T....................T................... 1 HE-N

24 ..............................................C....T.....A............. 1 SH

25 ..A...........................T....................T................... 1 JX-N

26 ..A.................................................................... 3 JX-J, HE-N,HE-S

27 ..A.......G............................................................ 1 HE-S

28 ..A......GG............................................................ 1 SH

29 ..A......GG...........................................................C 1 JX-N

30 ..A......GG.........C.................................................. 1 SH

31 .C........G.........C.................................................. 1 JX-J

32 ..A....T..G............................................................ 4 HE-N, HE-S, 2SH

33 ..A....T..G...........................................................C 1 GX

34 ..A....T..G.......................C.................................... 1 JX-J

35 ....................................C.................................. 1 JX-N

36 .......T............................C.................................. 4 HE-N, JX-J,JX-N, SH

37 .......T............................C................................C. 12 6SC-N,3SC-C, 3GX

38 .......T............................C....T............................. 1 SH

39 .......T.........C..................C.................................. 1 GX

40 ...G...T............................C................................C. 1 GX

41 .......T............C...............C................................C. 1 SC-N

42 .......T...........T................C................................C. 1 SC-N **CII**

43 .......T.............C..............C................................C. 1 SC-N

44 .......T............................C.....G..........................C. 1 SC-C

45 .......T....................A.......C.................................. 1 HE-N

46 .......T............................C....................A............. 1 SC-N

47 .......T............................C....................A...........C. 1 GX

48 ....................C...............C....................A............. 1 GX

49 ..........G.........................C....................A............. 1 SH

50 ..........G....................G.........................A............. 1 SH

51 ..........G..G...........C.....G....................................... 2 HE-N, HE-S

52 .........................................................T............. 8 HA,2HE-S,JX-N,2SC-N,2YN

53 ....C............G..A...........T........C.........T.....T.......T..... 1 HE-S

54 ...................................T....G................T..........A.. 4 4YN

55 ....................C........G...........................T............. 1 HE-S

56 ............G................................T...........T............. 2 SC-N,YN

57 .C.....................G..........C......................T..........A.. 1 HA

58 .C............G..........................................T............. 3 3YN

59 ......G.......G.......A..................................T............. 1 YN

60 ..A......................................................T............. 1 JX-N

61 ..A..............................GC......................T............. 1 JX-J

62 .........................................................T...........C. 2 HE-N, JX-N

63 ..A......................................................T...........C. 1 HA

64 ....................C.....................G..............T...........C. 1 JX-N

65 ....................C....................................TC..........C. 1 SH

66 ...............T..................................G......T...........C. 1 HA

67 ...............T....C...........G.................G......T...........C. 1 HA

68 .................................G.......................T............. 1 HE-S **CIII**

69 .................................G.....G.................T............. 1 SH

70 .................................G.......................TC.....C...... 1 JX-J

71 ...........G....................TG.......................T............. 3 2HE-S, SH

72 ...........G............C.......TG.......................T............. 1 HE-S

73 ........G..G....................T...C....C......CA.....GCT...........C. 1 HE-S

74 ...........G....................T...C....C.G...CCA......CT............. 1 SC-N

75 .....C.....G....................T...C.T..CCG.T..CA......CT............. 1 HE-S

76 .....C.....G....................T...C.T..CC..T..CA.T..G.CT............. 1 JX-N

77 .....C.....G....................T...C.T..CC.CT..CA.T..G.CT............. 3 HA, 2YN

78 .....C....GG....................T...C.T..CC.CT..CA.T..G.CT............. 7 7YN

79 .....C.T...G....................T...C.T..CC..T..CA......CT............. 1 SH

80 .....C.....G....................T...C.T..CG..T..CA....G.CT............. 1 JX-N

81 .....C.....G......C.............T...C.T..CC..T..CA.T..G.CT............. 1 SC-N

82 .....C.....G....G..............GT...C.T..CC..T..CG......CT............. 1 JX-J

83 .....C.....G.....C..............T...C.T..CC..T...A....G.CT...A......... 1 SH

84 C....C.....G....................T...C.T...C..T..CA.T..G.CT.G........... 1 HA

H= Haplotype number, n= Haplotype frequency, dot indicate identical with above reference, for

Population abbreviations see table 1 Position of amino acids replacement also bold at main reference. C = cluster
